# Severe Capnocytophaga Canimorsus Purpura Fulminans After a Cat Bite in an Asplenic Patient: Diagnostic and Therapeutic Challenges

**DOI:** 10.7759/cureus.84693

**Published:** 2025-05-23

**Authors:** Scott Vella Sorensen, Muhammad Khudayar, Humnah Khudayar

**Affiliations:** 1 School of Medicine, Frank H. Netter MD School of Medicine at Quinnipiac University, North Haven, USA; 2 Medicine, Shifa College of Medicine, Islamabad, PAK; 3 Pulmonary and Critical Care Medicine, MidState Medical Center, Meriden, USA

**Keywords:** asplenia, capnocytophaga canimorsus, cat bite infection, disseminated intravascular coagulation, multiorgan failure, purpura fulminans, septic shock, zoonotic infections

## Abstract

*Capnocytophaga canimorsus*, a gram-negative bacterium often found as part of the oral flora of dogs, can cause life-threatening infections in immunocompromised patients, usually secondary to a dog bite or scratch. This case report describes a life-threatening *Capnocytophaga *infection causing bacteremia and severe septic shock with multiple organ dysfunction in an asplenic man who was bitten by a cat.

We present a case of a 53-year-old asplenic man who developed severe septic shock, disseminated intravascular coagulation (DIC), and purpura fulminans following a minor cat bite. Despite prompt treatment with broad-spectrum antibiotics, his condition worsened to multiorgan failure. He required hemodialysis, mechanical ventilation, and management of acute vascular thrombosis, which led to prolonged intensive care. Diagnosis of the causative pathogen was initially delayed due to negative cultures, but microbial cell-free DNA testing ultimately identified *C. canimorsus*. Targeted therapy with meropenem led to eventual stabilization and improvement. This case highlights the pathogen’s immune evasion mechanisms and the importance of early empirical antibiotic coverage in at-risk patients. It also highlights the importance of thorough history-taking when suspecting unusual disease presentation, as this can lead to a timely diagnosis of a catastrophic illness.

Given the organism's slow growth in cultures and emerging resistance patterns, clinicians should be aware of the prevalence of *C. canimorsus *following dog or cat bites in immunocompromised patients to improve outcomes in severe infections.

## Introduction

*Capnocytophaga canimorsus* is a gram-negative, encapsulated rod that is part of the normal flora in the oral cavity of cats and dogs. It can be associated with fatal infection, usually secondary to a dog bite or scratch in an immunocompromised patient [1]. Patients particularly at risk are asplenic individuals, due to the spleen's specialized role in eliminating encapsulated bacteria, as well as those with alcohol use disorder, cirrhosis, and malignancies [2].

However, patients who are not immunocompromised can also have severe outcomes, especially with improper wound cleaning and care [3]. The exact incidence is unknown but is estimated to be 0.5-0.7 cases per million individuals each year [4]. The mortality rate varies depending on the severity of infection and associated risk factors and can be as low as 5% in meningitis, 26%-36% in bacteremia, and as high as 60% in septic shock [4]. The lower mortality rate in meningitis may be due to the pathogen’s slower progression to the meninges, which allows the immune system more time to mount an effective response [5].

A history of dog bites is reported in approximately 60% of cases, while dog scratches, licking, or close exposure to dogs are present in 24% of patients [5]. Infections secondary to cat exposure are rare and estimated to be ~3% of cases [5]. Although a history of splenectomy or alcohol abuse is associated with more than 35% of cases per a recent review, nearly 40% of cases are present in immunocompetent patients [5].

The bacterium is slow-growing on cultures with a mean incubation time of six days and may take up to 14 days before colonies become visible, often outside the standard cutoff of four to five days of incubation for growing most organisms [6]. Extended incubation time is often necessary, which can fall outside the normal microbiology laboratory protocol. As standard blood cultures often do not show the organism, confirmatory diagnosis can often be delayed. This is especially harmful in an infection that can quickly progress to fulminant septicemia with shock, purpura, disseminated intravascular coagulation (DIC), and multiorgan failure. Furthermore, there is currently a lack of standardized methods for susceptibility testing due to the slow growth, as well as few comparative studies of antibiotic treatment protocols.

*C. canimorsus* secondary to a cat bite is rarely described in the literature, to the best of our knowledge. Here, we present a patient with asplenia who presented with overwhelming infection and purpura fulminans with consumptive coagulopathy secondary to* C. canimorsus *from a cat bite.

## Case presentation

A 53-year-old man with a prior remote history of splenectomy due to gastric cancer was brought to the emergency room by his wife with a two-day history of worsening subjective fevers, chills, diarrhea, and lethargy with decreasing activity and mental acuity. On the morning of the presentation, the patient developed sudden-onset, rapid, and progressive purplish discoloration on his face, arms, back, abdomen, and legs, which was without pain or pruritus. History was notable for a small cat bite on his finger. He also endorsed heavy alcohol use on a nightly basis.

**Figure 1:**
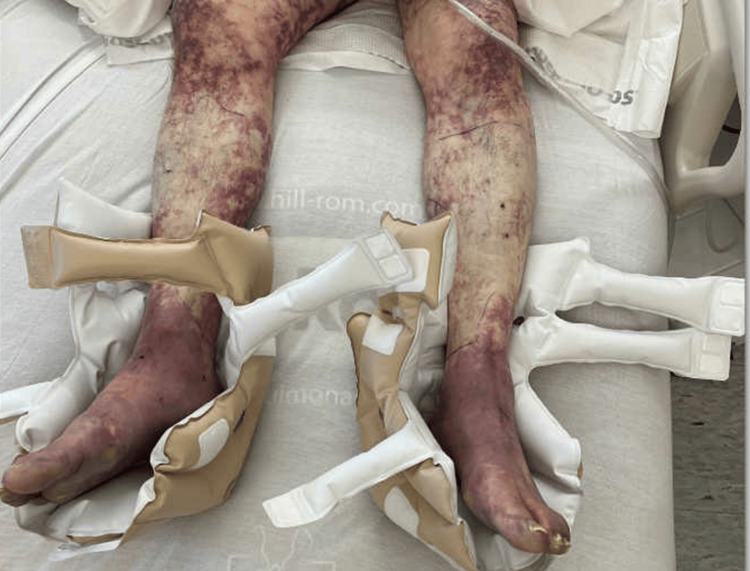
Lower extremities of the patient at the time of presentation

In the emergency department, he was afebrile but became progressively confused and lethargic. He had tachypnea, tachycardia, and refractory shock requiring vasopressors. Physical exam was notable for diffuse petechiae and purpura across his face, torso, and upper and lower extremities bilaterally (Figure 1). There were no signs of meningismus with negative Kernig’s and Brudzinski’s tests on physical examination.

On Day 0, labs showed severe abnormalities with a severe anion gap metabolic acidosis, lactic acidosis, leukocytosis, severe thrombocytopenia, coagulopathy, and transaminitis (Table 1). Following infectious disease consultation, he was started on ceftriaxone, vancomycin, azithromycin, and atovaquone to cover for gram-negative rods (including *Pasteurella *and *Capnocytophaga*), encapsulated bacteria (including *Neisseria spp*, *Streptococcus spp,* and *Staphylococcus spp*), and tick-borne pathogens (including *Babesia*).

**Table 1:** Laboratory results on the day of admission WBC: White blood cell count; Na: Sodium; K: Potassium; BUN: Blood urea nitrogen; AST: Aspartate transaminase; ALT: Alanine transaminase; LDH: Lactate dehydrogenase; CK: Creatine kinase; PT: Prothrombin time; PTT: Partial thromboplastin time; (H) indicates a high value compared to reference range; and (L) indicates a low value compared to reference range.

Test	Observed Values	Reference Ranges
WBC	29.7 x 10^3^/µL (H)	4.0-11.0 x 10^3^/µL
Hemoglobin	13.7 g/dL	13.0-17.7 g/dL
Platelets	22 x 10^3^/µL (L)	150-450 x 10^3^/µL
Na	136 mmol/L	136-145 mmol/L
K	5.0 mmol/L	3.4-5.3 mmol/L
Bicarbonate	14 mmol/L (L)	22-23 mmol/L
Anion gap	23 (H)	4-16
BUN	45 mg/dL (H)	8-21 mg/dL
Creatinine	4.7 mg/dL (H)	0.5-1.3 mg/dL
Calcium	7.4 mg/dL (L)	8.7-10.5 mg/dL
AST	3,001 U/L (H)	10-55 U/L
ALT	1,398 U/L (H)	10-55 U/L
Alkaline phosphatase	134 U/L (H)	45-128 U/L
Lipase	845 U/L (H)	13-60 U/L
Lactic acid	10.6 mmol/L (H)	0.5-1.9 mmol/L
Phosphorus	6.3 mg/dL (H)	2.7-4.5 mg/dL
LDH	3,263 U/L (H)	120-260 U/L
CK	3,368 U/L (H)	24-204 U/L
PT	26.9 seconds (H)	10.0-13.5 seconds
PTT	78 seconds (H)	25-35 seconds
Fibrinogen	102 mg/dL (L)	148-435 mg/dL
D-dimer	66,505 ng/mL DDU (H)	<230 ng/mL DDU

Blood cultures, sputum cultures, a viral panel (including Adenovirus, SARS-CoV-2, Influenza A/B, and Respiratory Syncytial Virus), a tick-borne illness panel (*Anaplasma*, *Ehrlichia*, and *Babesia*), fungal culture, and aspergillus antigen were all sent and eventually turned out negative.

**Figure 2:**
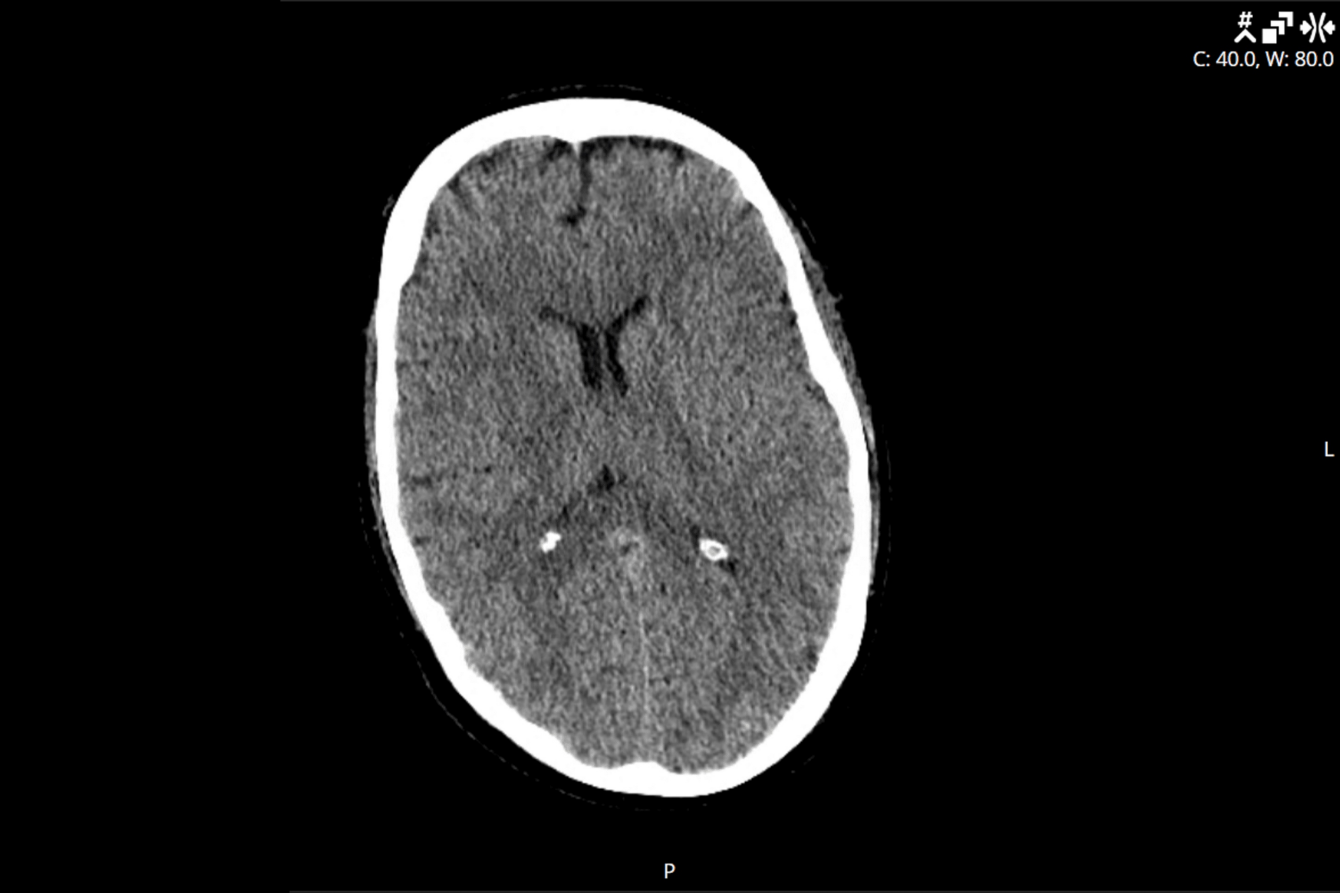
Noncontrast axial CT imaging of the brain with normal findings CT: Computed tomography.

**Figure 3:**
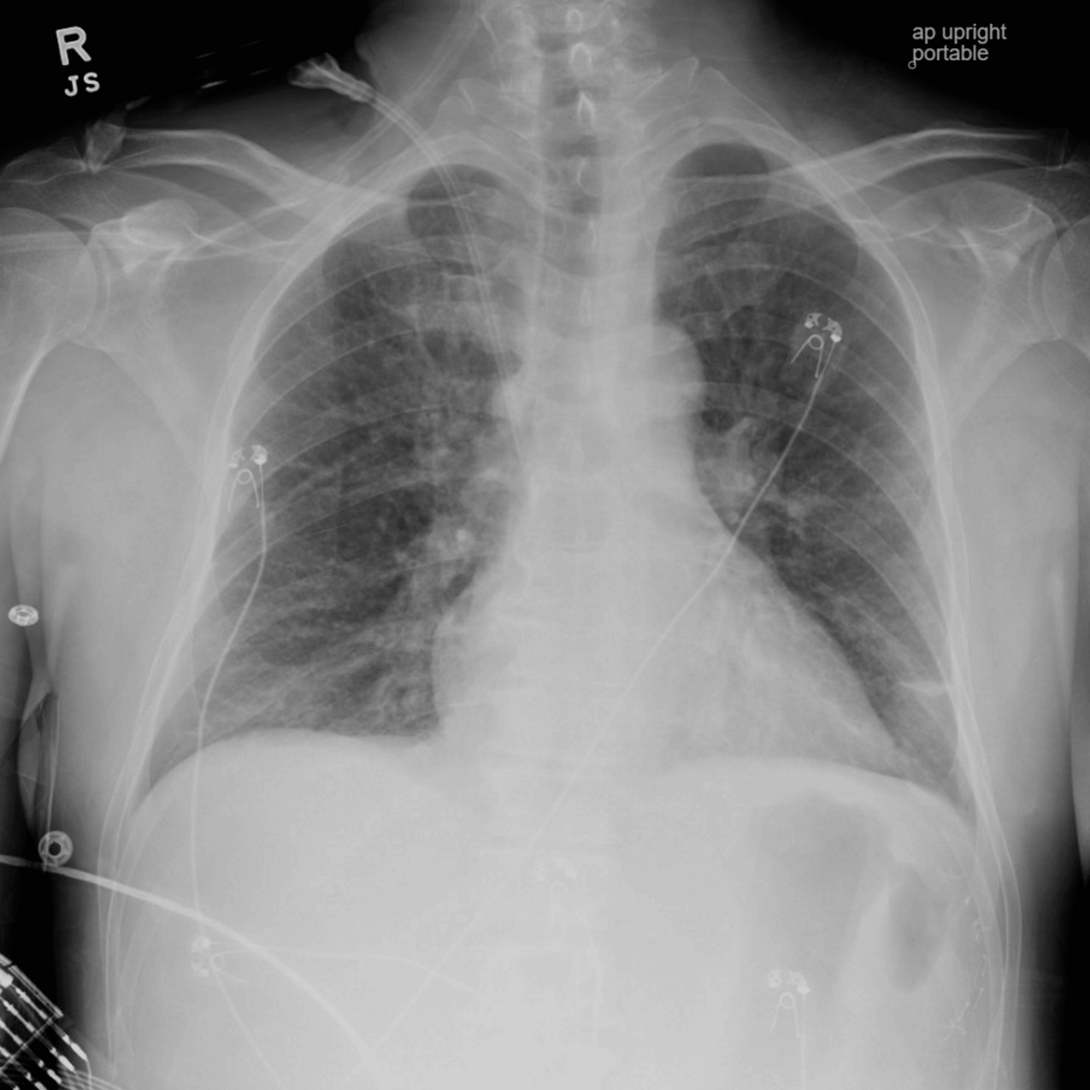
Chest X-ray at admission showing bilateral lower lobe atelectasis

**Figure 4:**
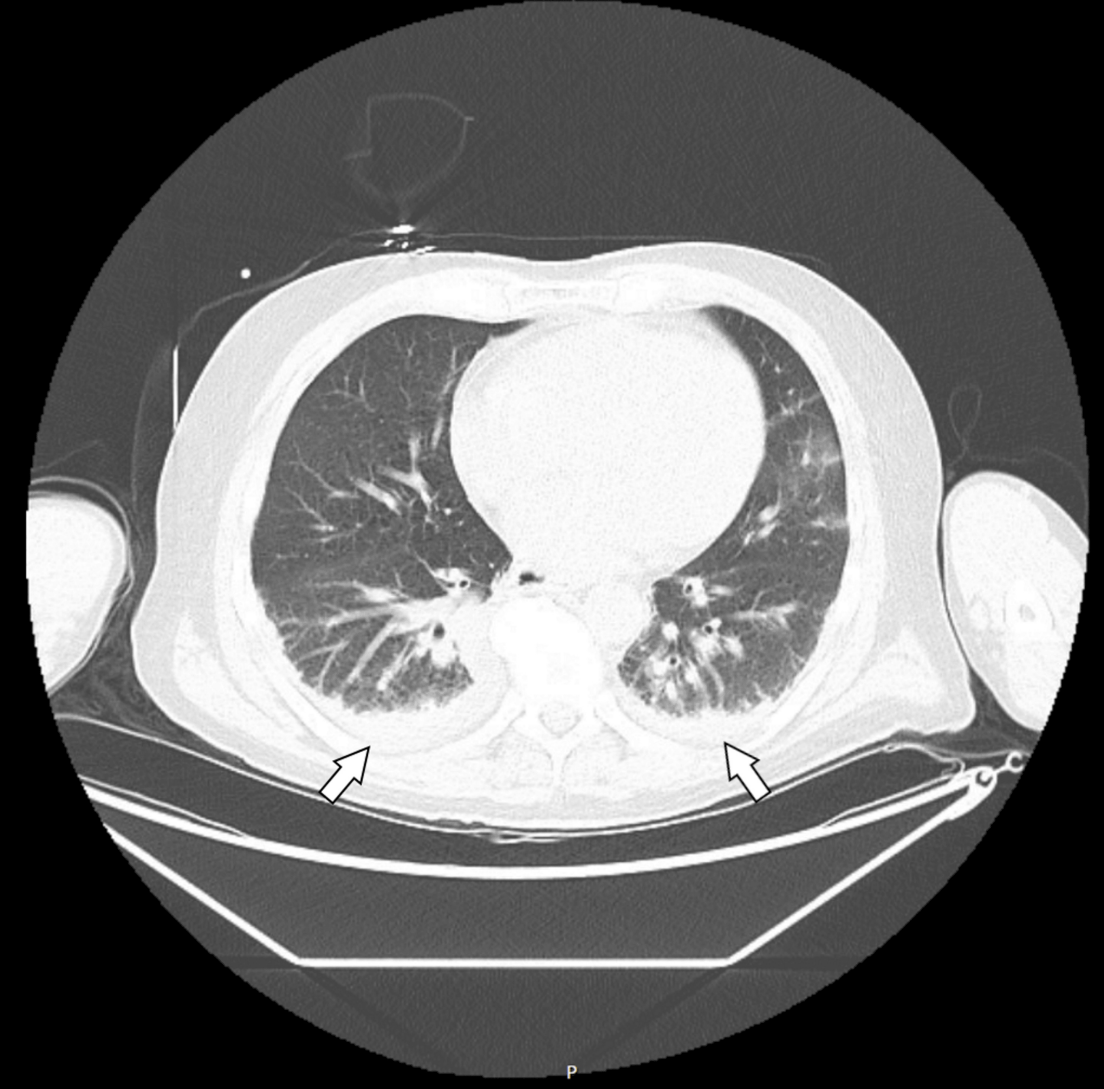
CT scan of the chest at admission showing bilateral lower lobe atelectasis and pleural effusions The arrows point to the pleural effusions. CT: Computed tomography.

Lumbar puncture was deferred due to profound coagulopathy as evidenced by both his hemorrhagic rash and abnormal clotting studies. Computed tomography (CT) imaging of the head was unremarkable (Figure 2). X-ray of the chest and CT of the chest and abdomen were consistent with bilateral lower lobe atelectasis and small pleural effusions (Figures 3, 4). Platelets and fresh frozen plasma (FFP) were administered, and he was transferred to the intensive care unit (ICU) for management of severe septic shock, DIC, purpura fulminans, and multiple organ dysfunction syndrome.

Day 1

**Figure 5:**
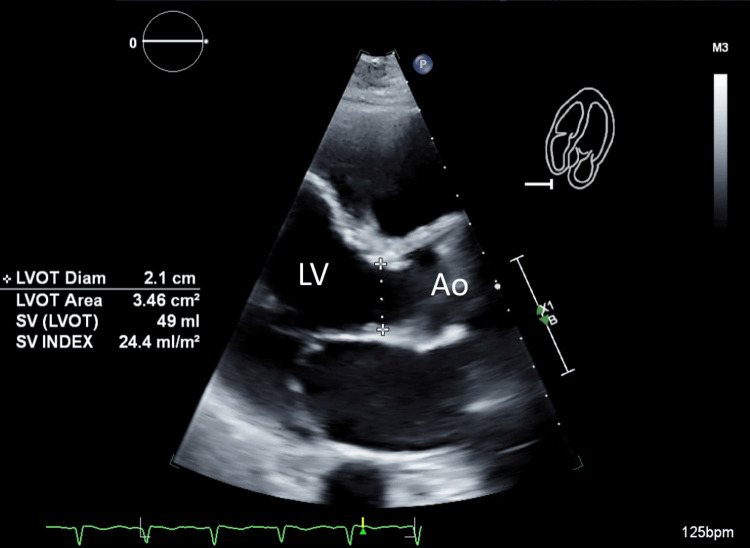
Transthoracic echocardiography (TTE) images showing left ventricular outflow tract Ao: Aorta; LV: Left ventricle; LVOT: Left ventricular outflow tract; SV: Stroke volume.

A hemodialysis catheter was placed, and dialysis was initiated secondary to renal failure with anuria and severe metabolic acidemia. Cryoprecipitate and platelet transfusion were administered due to persistent coagulopathy. Transthoracic echocardiogram (TTE) showed a depressed ejection fraction (EF) at 34%, with no evidence of regional wall motion abnormalities, valvular dysfunction, or vegetation (Figure 5).

Day 2

The patient developed hypoxic respiratory failure requiring intubation and mechanical ventilation. A platelet transfusion was given due to persistent thrombocytopenia.

Days 3-5

**Figure 6:**
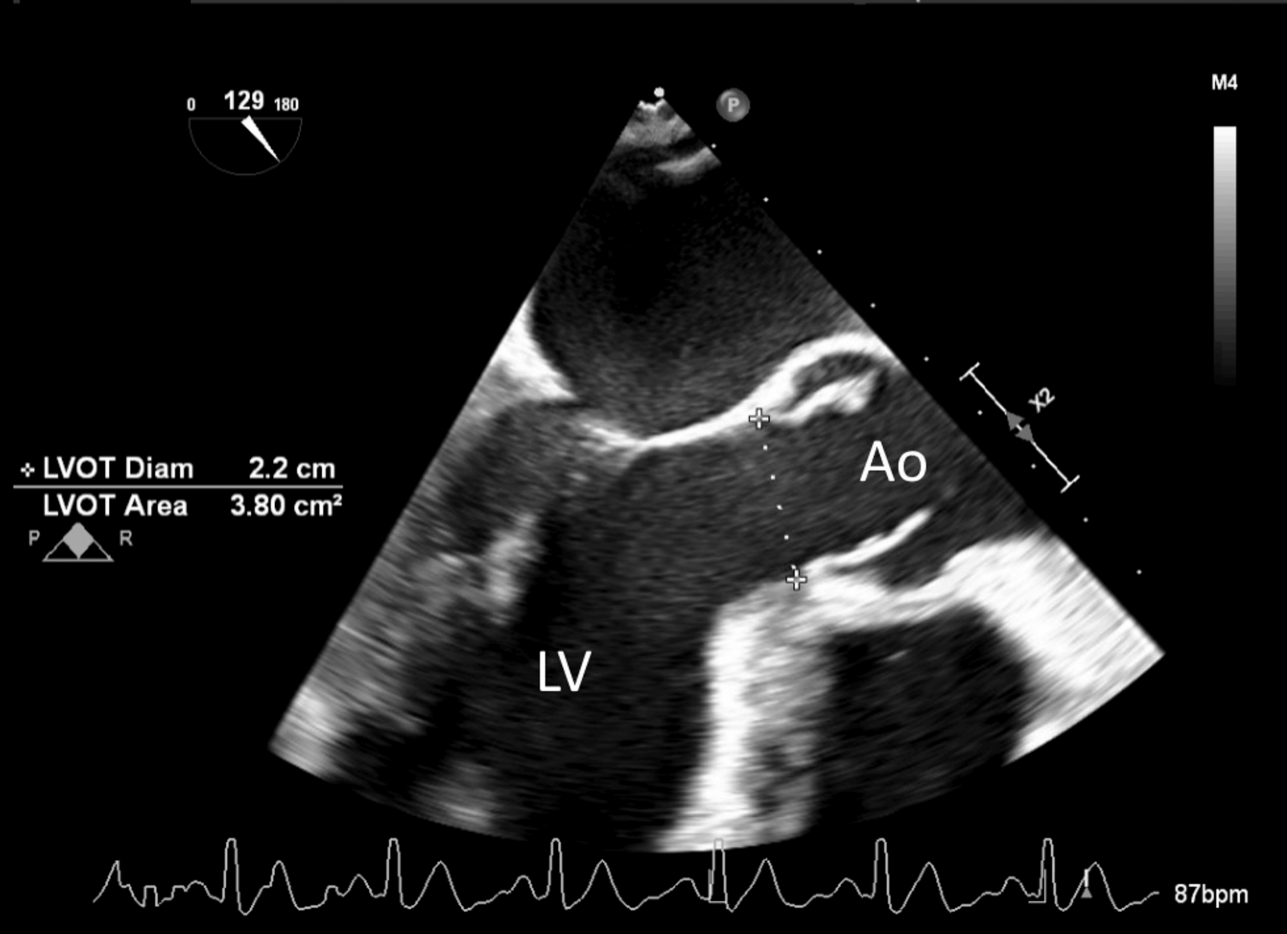
Transesophageal echocardiography (TEE) images showing left ventricular outflow tract without vegetations Ao: Aorta; LV: Left ventricle; LVOT: Left ventricular outflow tract.

Blood cultures, urine cultures, sputum cultures, viral panels, fungal panels, and tick-borne panels were persistently negative on testing. Thus, Karius cell-free DNA testing was sent out due to suspicions of an as-of-yet undetected fastidious organism. Due to the presence of a new-onset heart murmur on physical exam, a transesophageal echocardiogram (TEE) was performed to evaluate for infective endocarditis. The TEE showed an EF of 42% with no evidence of valvular vegetations, which ruled out endocarditis (Figure 6).

Antibiotics were updated to IV meropenem, IV penicillin G, and doxycycline with continued vancomycin with the intent of covering potential *Pasteurella spp*, *Capnocytophaga spp*, and *Rickettsia rickettsii *infections. Azithromycin was discontinued due to negative *Legionella* antigen testing.

Days 6-11

Multiple red blood cell (RBC) transfusions were required due to persistent and worsening anemia. Volume status improved on dialysis for consistent anuria, and spontaneous breathing trials were started successfully. *Rickettsia rickettsii*, dengue virus, and viral hepatitis serologies were negative. Karius cell-free DNA came back positive for* C. canimorsus*, and antibiotics were narrowed to a 21-day course of meropenem.

Days 12-15

Ongoing bleeding and sloughing of tissue in the patient's oral mucous membranes prompted ear, nose, and throat (ENT) specialists to perform elective tracheostomy, with eventual weaning to a tracheostomy mask.

Days 16-22

Purpuric rash showed signs of resolving with developing dry necrosis on fingertips and toes which was allowed to demarcate following the vascular surgery team's recommendations. The patient started making urine after a diuretic challenge. Blood urea nitrogen (BUN) and creatinine continued to trend downward, and dialysis was discontinued.

Day 23

**Figure 7:**
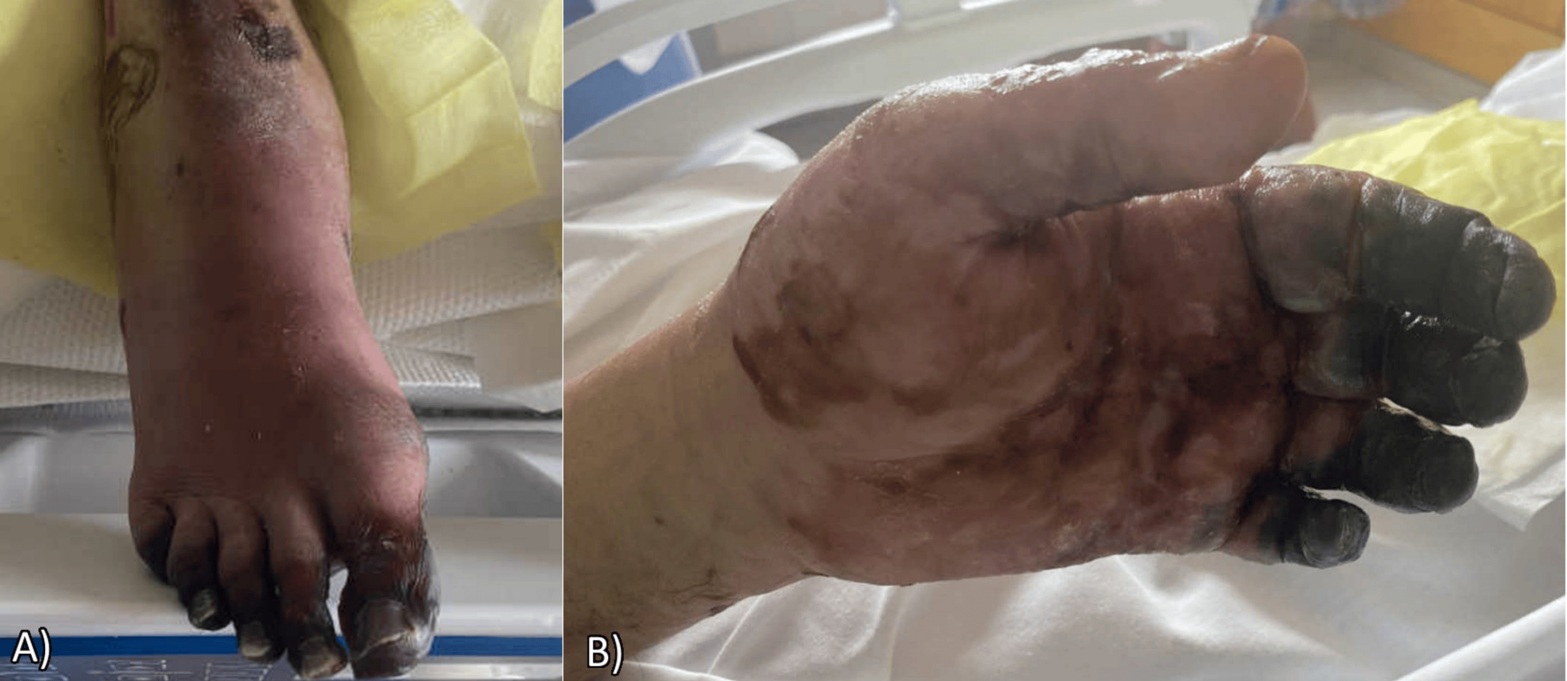
Continued demarcation of toes (A) and fingertips (B) before discharge from ICU

He was discharged to acute rehabilitation for further recovery. On discharge, his purpuric rash had mostly improved with continued demarcation and dry necrosis of fingertips and toes (Figure 7), which eventually required bilateral below-knee amputations and multiple finger amputations bilaterally. His BUN, creatinine, and liver enzymes had returned to baseline at discharge, and his *C. canimorsus* infection had resolved.

## Discussion

*C. canimorsus* is a gram-negative, facultatively anaerobic, encapsulated rod that can wreak havoc on the immune system. It has been shown to survive being engulfed by mouse macrophages [7]. The bacteria multiply within the macrophages, and electron microscopy shows the bacteria within the macrophage vacuoles. Cytokine activity and cell signaling pathways typically used by the macrophages to stimulate an immune response are downregulated or turned off [7]. This enables *C. canimorsus *to hide from the immune system for a longer period, while it multiplies undetected. *C. canimorsus *also appears to be resistant to complement and can kill and even thrive on polymorphonuclear leukocytes by deglycosylating host glycans [8].

Infections caused by *C. canimorsus* are reported far less frequently from cat bites than from dog bites. In reports that describe infection with *C. canimorsus *following cat exposure, presentations varied from severe diarrheal illness to multiorgan failure and DIC, similar to what was seen in the patient presented here [9,10]. *Purpura fulminans*, rapidly progressing cutaneous hemorrhage and necrosis, has been reported as a common finding from *C. canimorsus *infection*.* The mechanism is thought to involve a hyper-coagulable state secondary to the consumption of protein C, protein S, and antithrombin III by bacterial endotoxin [11]. This environment leads to the development of thromboses and DIC. Initially, petechial rashes develop and may evolve into purpuric lesions as the infection worsens, eventually culminating in necrosis and gangrene. Eventual amputation following the resolution of the infection has also been described.

Clinicians should remain vigilant for the possibility of *C. canimorsus* in patients with a suitable history of dog or cat exposure who present with a petechial or purpuric rash without meningeal signs. *C. canimorsus* is slow-growing and may not show up in blood cultures as shown in our case described here. Its mean incubation time of six days means that laboratories should be informed if the bacteria is suspected and that cultures may require an extended incubation time of at least six to eight days before being reasonably declared sterile [6]. Unfortunately, there is also a lack of standardized guidelines for susceptibility testing, which is challenging to perform due to the organism's slow growth rate in culture.

Falsely negative blood cultures and pathogen panels can provide mistaken reassurance that there is no underlying infectious etiology, which could lead to premature discontinuation of antibiotics and pursuit of alternative diagnoses. While testing microbial cell-free DNA is useful for confirming the diagnosis, it can also take a considerable amount of time to result. Therefore, microbial cell-free DNA should be considered at an earlier time point in patients with severe illness following animal exposure. Additionally, coverage of *C. canimorsus* should be ensured if the diagnosis is uncertain.

Currently, there is a relative lack of randomized studies and guidelines regarding the best antibiotic therapy and duration of treatment for *C. canimorsus* [12]. Many strains are susceptible to beta-lactam antibiotics, but the spread of beta-lactamase-producing strains has been described along with potential resistance to ceftriaxone therapy, with these resistances being more common among *Capnocytophaga *species other than *C. canimorsus* [13]. Currently, nearly all *Capnocytophaga *species including *C. canimorsus *are sensitive to beta-lactam antibiotics when used in combination with beta-lactamase inhibitors (amoxicillin-clavulanate, ampicillin-sulbactam, and piperacillin-tazobactam) or a carbapenem (imipenem and meropenem), making them an effective empirical treatment, especially in asplenic patients [12]. Susceptibility to cephalosporins, penicillins, quinolones, aminoglycosides, and vancomycin varies widely, making these antibiotics less than ideal for empiric treatment of *Capnocytophaga *infections* *[12]. Finally, the duration of treatment should depend on the severity of the infection and response to treatment.

Despite the lack of standardized trials and guidelines, the prognosis in septic patients who receive early empirical treatment with a carbapenem or a beta-lactam/beta-lactamase inhibitor combination for several weeks is generally favorable. Additionally, amoxicillin-clavulanate is an appropriate antibiotic prophylaxis in patients with a dog or a cat bite, especially those who are immunocompromised, as this provides coverage for the most frequently isolated pathogens from dog and cat bites, such as *Pasteurella spp. *and *Capnocytophaga spp.* [14].

## Conclusions

Although *C. canimorsus* is typically associated with dog bites and scratches, it can show up after exposure to cats, as seen in this case, and should be considered along with more common pathogens such as *Pasteurella multicoda* and *Bartonella henslae*. Increasing awareness of this pathogen among clinicians can help ensure timely intervention and improve outcomes in both typical and atypical presentations following animal exposure, especially given its slow-growing nature in blood cultures. Prompt and aggressive wound care after animal bites is recommended, along with antibiotic prophylaxis in immunocompromised patients, to prevent infection and reduce morbidity and mortality.
